# Computational Analysis of the Predicted Evolutionary Conservation of Human Phosphorylation Sites

**DOI:** 10.1371/journal.pone.0152809

**Published:** 2016-04-05

**Authors:** Brett Trost, Anthony Kusalik, Scott Napper

**Affiliations:** 1 Department of Computer Science, University of Saskatchewan, Saskatoon, Saskatchewan, Canada; 2 Vaccine and Infectious Disease Organization, University of Saskatchewan, Saskatoon, Saskatchewan, Canada; 3 Department of Biochemistry, University of Saskatchewan, Saskatoon, Saskatchewan, Canada; University of Rome Tor Vergata, ITALY

## Abstract

Protein kinase-mediated phosphorylation is among the most important post-translational modifications. However, few phosphorylation sites have been experimentally identified for most species, making it difficult to determine the degree to which phosphorylation sites are conserved. The goal of this study was to use computational methods to characterize the conservation of human phosphorylation sites in a wide variety of eukaryotes. Using experimentally-determined human sites as input, homologous phosphorylation sites were predicted in all 432 eukaryotes for which complete proteomes were available. For each pair of species, we calculated phosphorylation site conservation as the number of phosphorylation sites found in both species divided by the number found in at least one of the two species. A clustering of the species based on this conservation measure was concordant with phylogenies based on traditional genomic measures. For a subset of the 432 species, phosphorylation site conservation was compared to conservation of both protein kinases and proteins in general. Protein kinases exhibited the highest degree of conservation, while general proteins were less conserved and phosphorylation sites were least conserved. Although preliminary, these data tentatively suggest that variation in phosphorylation sites may play a larger role in explaining phenotypic differences among organisms than differences in the complements of protein kinases or general proteins.

## Introduction

Protein kinases play a critical role in regulating cellular processes by catalyzing the phosphorylation of amino acid residues (usually serine, threonine, or tyrosine), which may activate, deactivate, or change the activity of the target protein [1, 2]. Most eukaryotic genomes encode a large number of protein kinases; for example, the human genome encodes more than 500 [2], while the *Arabidopsis thaliana* genome encodes more than 1000 [3]. The complement of protein kinases in a species is called its kinome, while the complement of phosphorylation sites—the residues phosphorylated by protein kinases—is called its phosphoproteome.

Given their functional importance, as well as their integral role in determining the phenotype of a given organism, several studies have been devoted to characterizing the evolution and conservation of phosphorylation sites. For example, Rinschen et al. discovered that cross-species comparisons of phosphorylation site data can provide insight into the architecture of specific signaling systems [4], while Kim et al. compared known phosphorylation sites in several evolutionarily-diverse organisms, and found several sites in human that appear to have arisen since the divergence of humans and chimpanzees [5]. Moses et al. examined the evolution of sites phosphorylated by cyclin-dependent kinases in proteins comprising the pre-replicative complex (a set of interacting proteins involved in DNA replication) in a number of closely- and distantly-related organisms [6]. They found that although pre-replicative complex components often contained clusters of cyclin-dependent kinase consensus sites, the presence or position of individual sites were often poorly conserved, and that differences in these sites were associated with regulatory changes. Recently, Freschi et al. [7] performed a detailed study of the known human and mouse phosphoproteomes, and found that while approximately 87% of the phosphorylation sites that have been experimentally determined to exist in one species or the other were conserved at the sequence level, a much lower proportion were actually known to be phosphorylated in both species. The authors then analyzed how often these discrepancies can be attributed to factors like false negatives and false positives in experimentally identifying sites, as well as to non-functional sites. From this analysis, they estimated that approximately 5% of the sites that were conserved at the sequence level, but were known to be phosphorylated in only one of the two species, represented sites that were truly differentially regulated between the two species.

In another study, Jimenez et al. found that phosphorylation sites are less well-conserved in different organisms than would be expected given their functional significance [8], although other studies have shown that phosphorylation sites are better conserved than serine, threonine, and tyrosine residues in general [9, 10]. Nguyen-Ba et al. showed that mutations in yeast phosphorylation sites are highly constrained relative to surrounding sites in both structured and unstructured regions of proteins [11]. Gnad and co-authors [12] found that proteins that contain phosphorylation sites are more likely to be conserved across species than proteins in general, and also developed a web-based tool (PHOSIDA) that integrates evolutionary information about phosphorylation sites with a database of experimentally-determined phosphorylation sites and a phosphorylation site prediction algorithm. Another software program devoted to the evolution of phosphorylation sites is CPhos, which—based on the assumption that functional phosphorylation sites are more likely to be conserved than non-functional sites—aids the user in identifying functional phosphorylation sites by assessing the degree of conservation of a given site in a number of different organisms [13]. Finally, although the study of phosphorylation site conservation has typically been limited to eukaryotes, such studies have also been done in prokaryotes [14].

While the studies cited above have examined the evolution and conservation of phosphorylation sites in a limited number of organisms and/or for a limited number of protein kinases, to our knowledge there has not yet been a study characterizing the general conservation of human phosphorylation sites in a wide variety of species. Whereas protein kinases can be readily identified using sequence and structural homology, allowing the kinomes of different species to be characterized and compared relatively easily [15, 16], several challenges are associated with estimating the degree of phosphorylation site conservation in different species. First, there are far more phosphorylation sites in a species than protein kinases; for instance, the human proteome appears to contain more than 100,000 phosphorylation sites [17] versus only around 500 protein kinases [2]. Second, the number of known phosphorylation sites varies substantially depending on the species: while there are many known phosphorylation sites for some species (such as human, mouse, and *A. thaliana*), our knowledge of phosphorylation sites in other species ranges from sparse (e.g., approximately 500 in cattle) to almost nonexistent (e.g., honeybee) [17]. Third, the sequence-based identification of phosphorylation sites is more difficult than the sequence-based identification of protein kinases [18–20].

In this study, a methodology that addresses these challenges is used to characterize the degree of conservation of human phosphorylation sites among a wide variety of species. Phosphorylation site conservation is then compared to the conservation of both protein kinases and proteins in general. Given that fewer genomic changes are required for the gain or loss of a phosphorylation site as compared to the gain or loss of a protein kinase, we hypothesize that phosphorylation sites will be less conserved than protein kinases.

## Materials and Methods

### Data

Experimentally-determined human phosphorylation sites (*n* = 146306) were gathered from the online databases PhosphoSitePlus [17, 21, 22] and Phospho.ELM [23]. Of these sites, 8842 had one or more references describing the characterization of that site using low-throughput biological techniques; the remainder were determined using only high-throughput methods (typically mass spectrometry). We will denote the complete set of experimentally-determined human phosphorylation sites as *P*_*F*_ and the subset consisting only of sites with low-throughput references as *P*_*L*_. Complete eukaryotic proteomes (*n* = 432) were downloaded from UniProt [24] using the query “complete:yes ancestor:2759” (where 2759 is the taxonomic ID for eukaryotes).

### Measuring phosphorylation site conservation

The degree of phosphorylation site conservation between each pair of species *S*_*A*_ and *S*_*B*_ was assessed as follows. For a given phosphorylation site *i*, let *f*_*i*_(*S*) = 1 if the proteome of species *S* contains phosphorylation site *i*; otherwise, *f*_*i*_(*S*) = 0. Additionally, define the functions *E*_*i*_(*S*_*A*_, *S*_*B*_) and *B*_*i*_(*S*_*A*_, *S*_*B*_) as follows:

$$\begin{array}{c} E_{i}\left(S_{A},S_{B}\right)=\left\{\begin{array}{cc} 1 & \text{if}\;f_{i}\left(S_{A}\right)=1\;\text{or}\;f_{i}\left(S_{B}\right)=1\\ 0 & \text{otherwise} \end{array}\right. \end{array}$$

$$\begin{array}{c} B_{i}\left(S_{A},S_{B}\right)=\left\{\begin{array}{cc} 1 & \text{if}\;f_{i}\left(S_{A}\right)=1\;\text{and}\;f_{i}\left(S_{B}\right)=1\\ 0 & \text{otherwise} \end{array}\right. \end{array}$$

*E*_*i*_(*S*_*A*_, *S*_*B*_) is equal to 1 if either *S*_*A*_ or *S*_*B*_ (or both) contains phosphorylation site *i*. *B*_*i*_(*S*_*A*_, *S*_*B*_) is equal to 1 only if both *S*_*A*_ and *S*_*B*_ contain phosphorylation site *i*. Finally, we define *C*(*S*_*A*_, *S*_*B*_), which represents the number of phosphorylation sites present in both *S*_*A*_ and *S*_*B*_ divided by the number present in at least one of *S*_*A*_ and *S*_*B*_ (expressed as a percentage). Stated differently, the values *C*(*S*_*A*_, *S*_*B*_) represent the percentage of phosphorylation sites that are present in at least one of *S*_*A*_ and *S*_*B*_ that are present in both. Mathematically,

$$\begin{array}{c} C\left(S_{A},S_{B}\right)=100\times \frac{\sum_{i=1}^{p}B_{i}\left(S_{A},S_{B}\right)}{\sum_{i=1}^{p}E_{i}\left(S_{A},S_{B}\right)} \end{array}$$

where *p* is the number of phosphorylation sites examined. Greater values of *C*(*S*_*A*_, *S*_*B*_) indicate greater conservation, and vice versa.

As the number of experimentally-determined phosphorylation sites in many species is limited, it is difficult to accurately calculate the *f*_*i*_’s using experimental data. Thus, we calculated the *f*_*i*_’s by employing the online tool DAPPLE [25] to predict phosphorylation sites in the various species using experimentally-determined phosphorylation sites from human (the species with the greatest number of known phosphorylation sites). A brief description of DAPPLE is as follows. DAPPLE uses as input experimentally-determined phosphorylation sites from species other than the species for which predictions are being made (the “target species”). Each known phosphorylation site is represented as a 15-residue subsequence (peptide) of a full protein (with the phosphorylated residue in the center and 7 residues on either side), which is used as a BLAST query against the proteome of the target species. DAPPLE reports a number of pieces of information about each query peptide. Given that protein kinases recognize phosphorylation sites within the context of specific (but degenerate) sequence motifs, the most important piece of information given by DAPPLE is the number of sequence differences between the query peptide and its best match in the target proteome. If the number of sequence differences is small, then it is likely that the recognition pattern remains intact, and thus the residue in the matching peptide that corresponds to the phosphorylation site in the query peptide is considered a putative phosphorylation site. In contrast, if the number of sequence differences is large, then it is likely that the recognition pattern no longer exists, and thus the matching residue is not considered a putative phosphorylation site. For example, residue S53 in the protein with UniProt accession number Q8N0S6 is a known human phosphorylation site [17, 21, 22]. The 15-mer peptide with S53 at its center is RRKIPQCSQLQEDVD, which spans residues 46–60 in the full protein sequence. A BLAST search against the bovine proteome using this peptide as a query gives RRKIPQGSQLQEDVD as the best match, which is found in the bovine protein with accession number Q5EA18. This peptide comprises residues 48–62 of the full protein, and the phosphorylated residue is S55. Given that there is only one mismatch between the two sequences (C→G in position 7 of the peptides), it is likely that residue S55 in the bovine protein Q5EA18 is a phosphorylation site.

The *f*_*i*_’s were calculated from the DAPPLE output as follows. Let *Q*_*i*_ represent the 15-mer peptide corresponding to known human phosphorylation site *i*, and *H*_*i*, *S*_ denote its best match in species *S*. We set *f*_*i*_(*S*) = 1 if the number of sequence differences between *Q*_*i*_ and *H*_*i*, *S*_ was less than or equal to a threshold *T*; otherwise, *f*_*i*_(*S*) = 0. To select an appropriate value for *T*, we used previously-published data concerning how the number of sequence differences between a query 15-mer and its best match in the target proteome affects the likelihood that the best match is a known phosphorylation site [26]. In addition to *f*_*i*_, an alternative function *g*_*i*_ was defined in which *g*_*i*_(*S*) = 1 if the number of *non-conservative* sequence differences between *Q*_*i*_ and *H*_*i*, *S*_ is less than or equal to *T*, and 0 otherwise. A substitution was considered non-conservative if the entry for that substitution in the BLOSUM62 substitution matrix was less than zero. The usage of *f*_*i*_ or *g*_*i*_ is explained further at the end of this section.

After selecting an appropriate value for *T*, the values *C*(*S*_*A*_, *S*_*B*_) were calculated for each pair of species *S*_*A*_ and *S*_*B*_, and the results were compiled into a table (similarity matrix). As the number of species was 432, the number of possible pairs of species was (4322)=93096. A distance *D*(*S*_*A*_, *S*_*B*_) = 100−*C*(*S*_*A*_, *S*_*B*_) was also calculated for each pair, and the R function hclust was used to perform hierarchical clustering using these distances. Average linkage was used as the linkage method. The function *hc2Newick* from the ctc R package was used to convert the hierarchical clustering to Newick format. The Newick file was visualized using TreeGraph 2 [27].

Four different variations of the above procedure were performed. The differences between each variation relate to whether *f*_*i*_(*S*) or *g*_*i*_(*S*) was used to determine if species *S* contains phosphorylation site *i*, and whether the set of phosphorylation sites used was *P*_*F*_ or *P*_*L*_. Specifically, Method 1 used *f*_*i*_ and *P*_*F*_, Method 2 used *f*_*i*_ and *P*_*L*_, Method 3 used *g*_*i*_ and *P*_*F*_, and Method 4 used *g*_*i*_ and *P*_*L*_.

To determine how the values of *C*(*S*_*A*_, *S*_*B*_) derived using Method 1 compared to those derived using Methods 2, 3, and 4, the following procedure was used. The methods were compared both in terms of the degree of difference (i.e., how different are the results obtained when using Method 1 versus when using Method 2?) and the direction of difference (i.e., do phosphorylation sites appear to be more conserved or less conserved when using Method 1 versus when using Method 2?). Let *C*_1_(*S*_*A*_, *S*_*B*_), *C*_2_(*S*_*A*_, *S*_*B*_), *C*_3_(*S*_*A*_, *S*_*B*_), and *C*_4_(*S*_*A*_, *S*_*B*_) denote the value of *C*(*S*_*A*_, *S*_*B*_) obtained using Methods 1, 2, 3, and 4, respectively. To determine the *degree* of difference between Method 1 and Method 2, the value |*C*_1_(*S*_*A*_, *S*_*B*_)−*C*_2_(*S*_*A*_, *S*_*B*_)| was computed for all possible pairs (*S*_*A*_, *S*_*B*_), generating a list of 93096 values, with each value representing the degree of difference between the two methods for one pair of species. The mean, median, standard deviation, and range of these differences were then calculated, which together describe the overall degree to which the two methods differ in calculating phosphorylation site conservation. The degree of difference between Method 1 and Method 3 was then determined by calculating the values |*C*_1_(*S*_*A*_, *S*_*B*_)−*C*_3_(*S*_*A*_, *S*_*B*_)| and then determining the aforementioned statistical parameters, and similarly for Method 1 versus Method 4. To compute the *directionality* of the difference between Method 1 and Method 2, the same procedure as above was used except without taking the absolute value (that is, *C*_1_(*S*_*A*_, *S*_*B*_)−*C*_2_(*S*_*A*_, *S*_*B*_) was computed for each (*S*_*A*_, *S*_*B*_) instead of |*C*_1_(*S*_*A*_, *S*_*B*_)−*C*_2_(*S*_*A*_, *S*_*B*_)|), and similarly for Method 1 versus Method 3 and Method 1 versus Method 4.

### Measuring protein kinase conservation

To determine the degree to which protein kinases are conserved in different species, protein orthology data were used. As we are not aware of an orthologue database that includes all (or even most) of the 432 species whose proteomes were downloaded from UniProt, twenty species were selected that were present in the orthologue database OrthoMCL-DB [28]. These species represented a range of lineages (mammals: *Homo sapiens*, *Pan troglodytes*, *Canis lupus familiaris*, *Mus musculus*, and *Rattus norvegicus*; insects: *Drosophila melanogaster*, *Apis mellifera*, and *Anopheles gambiae*; fish: *Danio rerio* and *Tetraodon nigroviridis*; plants: *Arabidopsis thaliana*, *Oryza sativa*, and *Ricinus communis*; birds: *Gallus gallus*; arachnids: *Ixodes scapularis*; nematodes: *Caenorhabditis elegans*; others (single-celled organisms of different lineages): *Plasmodium falciparum*, *Saccharomyces cerevisiae*, *Chlamydomonas reinhardtii*, and *Trypanosoma vivax*). A list of orthologous groups was obtained from OrthoMCL-DB, where the entry for each group contained a list of the proteins from each species (if any) that were members of that group. Groups for which at least one of the member proteins contained at least one of the terms “tyrosine kinase”, “protein kinase”, or “serine/threonine kinase” were included in the analysis. The degree of protein kinase conservation was assessed using the same general framework as was used for determining phosphorylation site conservation. Specifically, *f*_*i*_(*S*) = 1 if there existed a protein kinase from species *S* in orthologous group *i*, and *f*_*i*_(*S*) = 0 otherwise. All of the other functions were defined analogously; in particular, *C*(*S*_*A*_, *S*_*B*_) represented the proportion of orthologous groups containing a protein kinase from at least one of *S*_*A*_ or *S*_*B*_ that contained a protein kinase from both.

### Measuring general protein conservation

To assess the general degree of protein conservation among the 20 species selected, the same procedure as described in the previous section was used, except all orthologous groups from OrthoMCL-DB were used, rather than just those containing proteins annotated as protein kinases.

## Results

### Determining an appropriate threshold *T*

To select an appropriate value for *T* (see the Materials and Methods section), we used the data presented in Table 3 of Trost et al. [26]. This table suggests that the more sequence differences between a 15-mer representing a known phosphorylation site and its best match in the target proteome, the lower the probability that the match represents a known phosphorylation site. Let *d* represent the number of sequence differences for which the probability that a match with that number of sequence differences is a known phosphorylation site is approximately half the probability of a match with zero sequence differences being a known phosphorylation site. We chose *T* to be equal to *d*. Although *d* differed somewhat depending on the species, Table 3 of Trost et al. [26] shows that *d* ≈ 6. Thus, we set *T* = 6.

### Measuring phosphorylation site conservation

The degree of phosphorylation site conservation *C*(*S*_*A*_, *S*_*B*_) was determined between each pair of species *S*_*A*_ and *S*_*B*_ for which a complete proteome was available (432 species). *C*(*S*_*A*_, *S*_*B*_) represents the percentage of phosphorylation sites present in at least one of *S*_*A*_ and *S*_*B*_ that were present in both. As described earlier, we performed four different variations of our procedure for determining the *C*(*S*_*A*_, *S*_*B*_) values. These variations were denoted Method 1, Method 2, Method 3, and Method 4. The differences between the four methods relate to the dataset of human phosphorylation sites (Methods 1 and 3 used the entire dataset of human phosphorylation sites, while Methods 2 and 4 used the subset of those sites that have been verified using low-throughput biological techniques) and in how phosphorylation site conservation was measured (Methods 1 and 2 used the number of sequence differences between a query 15-mer and its best match in the target proteome, while Methods 3 and 4 used the number of *non-conservative* sequence differences). The reason that Methods 2 and 4 used the subset of human phosphorylation site data verified using low-throughput techniques (rather than the full dataset of human phosphorylation sites) is that it has been suggested that a sizeable portion of sites identified only using discovery-mode mass-spectrometry experiments are non-functional and do not play a role in cellular signaling [17, 29], and because phosphorylation sites with known functions appear to be more evolutionarily conserved than sites with unknown function [9, 10]. Thus, restricting the analysis to sites that have been verified to be functional may provide a different picture of phosphorylation site conservation as compared to using all sites, including those identified only via mass spectrometry. The reason for performing the procedure by counting only non-conservative substitutions rather than all substitutions when determining the presence or absence of a given site (Methods 3 and 4) is that conservative substitutions surrounding a phosphorylation site may have a smaller impact on the ability of the kinase to recognize that site than non-conservative substitutions. In the following, we begin by describing the results obtained using Method 1. We then compare the results when using Method 1 to the results when using Methods 2, 3, and 4.

**Fig 1:**
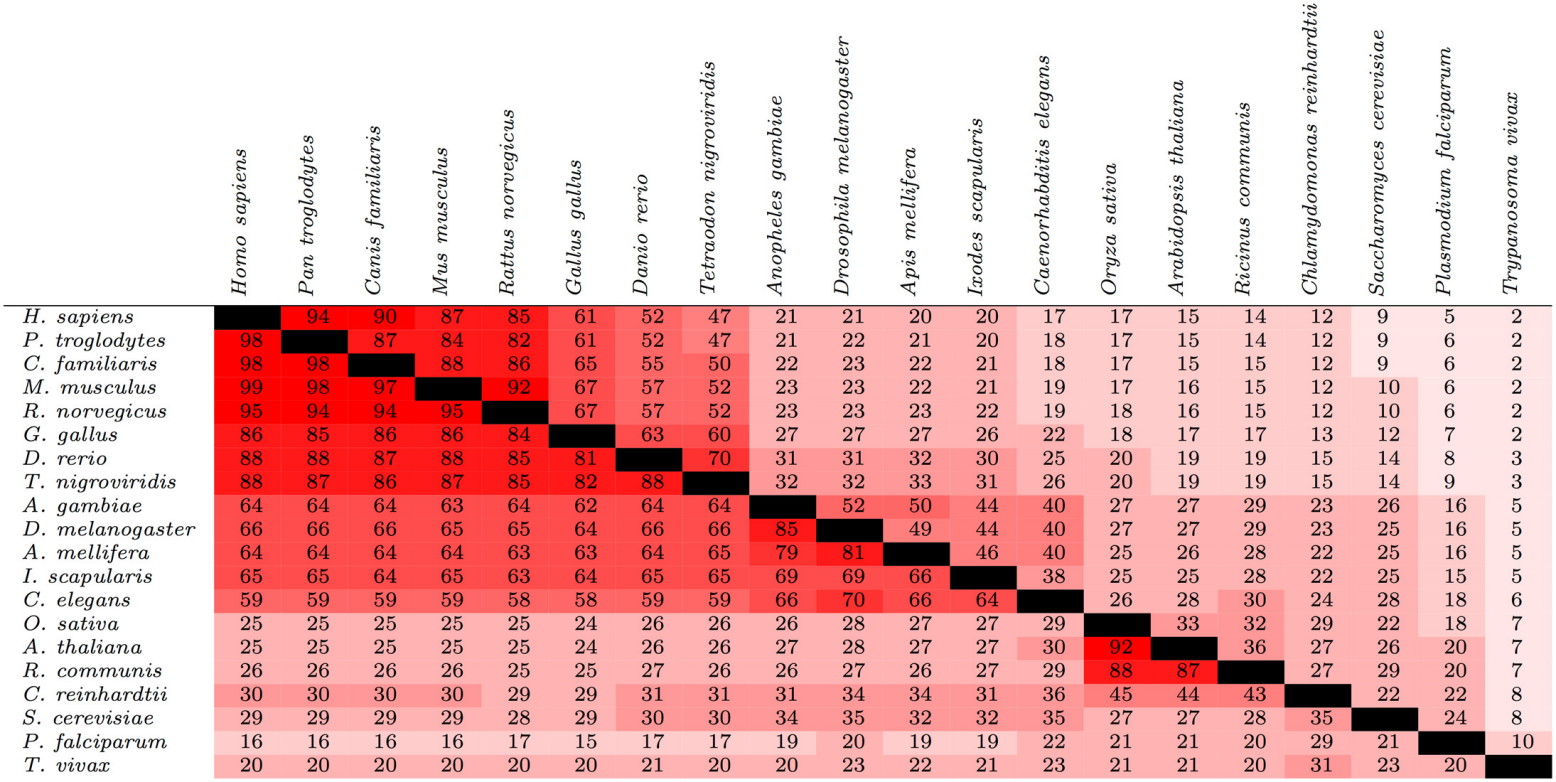
Conservation of phosphorylation sites (upper triangle) and protein kinases (lower triangle) among 20 species from a diverse range of lineages. For a given pair of species, the values represent the percentage of phosphorylation sites or protein kinases found in at least one of the two species that were found in both species (the values *C*(*S*_*A*_, *S*_*B*_) described in the text). The cells are colored based on the value within; the closer the value is to 100, the brighter the shade of red.

The upper triangle of Fig 1 contains the values of *C*(*S*_*A*_, *S*_*B*_) when using Method 1 for the 20 species described in the Materials and Methods section; S1 Table includes all 432 species. These conservation values appear to be consistent with prior studies; for instance, Freschi et al. examined the conservation of known phosphorylation sites in human and mouse, and found that 84% of the phosphorylation sites that had been experimentally determined in at least one of the two species were conserved at the sequence level [7]. Here, we report that 87% of experimentally-determined human sites were conserved at the sequence level in mouse (Fig 1).

**Fig 2:**
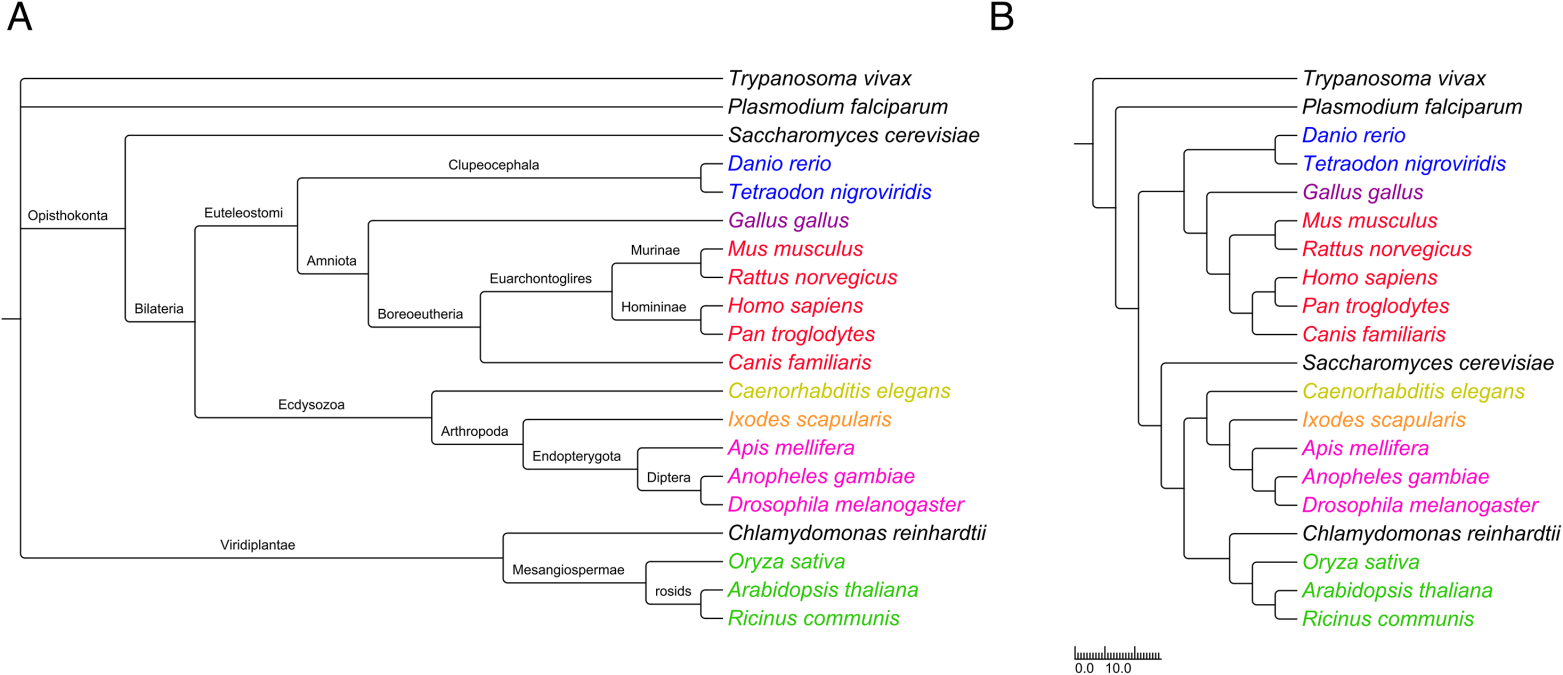
Comparison between the taxonomy of the 20 species described in the Materials and Methods section according to the National Center for Biotechnology Information (NCBI) Taxonomy Browser (panel A), and the dendrogram generated based on the phosphorylation site conservation of pairs of species (panel B). The scale applies to panel B only, with the branch lengths representing values of *C*(*S*_*A*_, *S*_*B*_). The species names are color-coded based on lineage: red, mammals; pink, insects; blue, fish; green, plants; purple, birds; orange, arachnids; yellow, nematodes; black, others (single-celled organisms of different lineages).

A dendrogram created using hierarchical clustering was generated using the distances *D*(*S*_*A*_, *S*_*B*_) for the 20-species subset, and was compared to the taxonomy for those species as given by the National Center for Biotechnology Information (NCBI) [30] Taxonomy Browser (Fig 2). Visually, the two trees are quite similar—most branches that are close to leaves have identical sets of descendants, although the branching patterns differ more closer to the root. To obtain a quantitative measure of the similarity of the two trees, we used the T-REX web server [31] to compute the Robinson and Foulds distance, which reflects the number of operations needed to convert one tree into another [32]. The calculated distance was 11; thus, relatively few operations are needed to transform the NCBI tree into the tree based on the conservation of human phosphorylation sites. A dendrogram based on the distances *D*(*S*_*A*_, *S*_*B*_) for all 432 species is given in S1 Fig.

Interestingly, the degree of phosphorylation site conservation appeared to differ depending on the residue being phosphorylated. Specifically, the average of all the *C*(*S*_*A*_, *S*_*B*_) values was 21.8 for Ser sites, 31.7 for Thr sites, and 28.7 for Tyr sites (all differences statistically significant with p-value≪0.001 according to a two-tailed t-test). S2–S4 Tables contain the values of *C*(*S*_*A*_, *S*_*B*_) for Ser, Thr, and Tyr sites, respectively. S5 Table contains the average value of *C*(*S*_*A*_, *S*_*B*_) for each species (averaged over all of the other species) for each phosphorylated residue.

Figs 1 and 2 show that the relative degree of phosphorylation site conservation among the different species was consistent with their known evolutionary relationships. For instance, Fig 1 shows that 94% of the phosphorylation sites in either *H. sapiens* and *P. troglodytes* were found in both (*C*(*S*_*A*_, *S*_*B*_) = 94), whereas *C*(*S*_*A*_, *S*_*B*_) = 21 for *H. sapiens* and *D. melanogaster*. The pair of species with the lowest degree of similarity included *H. sapiens* and the parasite *T. vivax*, with *C*(*S*_*A*_, *S*_*B*_) = 2.

To determine the degree of difference between the values of *C*(*S*_*A*_, *S*_*B*_) determined using Method 1 and the values obtained using Method 2, |*C*_1_(*S*_*A*_, *S*_*B*_)−*C*_2_(*S*_*A*_, *S*_*B*_)| was calculated for each pair (*S*_*A*_, *S*_*B*_), where *C*_1_(*S*_*A*_, *S*_*B*_) represents the value of *C*(*S*_*A*_, *S*_*B*_) calculated using Method 1 and *C*_2_(*S*_*A*_, *S*_*B*_) represents the value of *C*(*S*_*A*_, *S*_*B*_) calculated using Method 2. Let *E* represent the list of differences for all pairs (*S*_*A*_, *S*_*B*_). Some simple descriptive statistics (mean, median, range, and standard deviation) were then calculated for the values in *E*. The same procedure was then used to characterize how the values of *C*(*S*_*A*_, *S*_*B*_) differ between Method 1 and Method 3, and between Method 1 and Method 4. The results of this analysis are given in Table 1, which shows that the degrees of difference between Method 1 and Methods 2, 3, and 4 were relatively small. Specifically, the mean difference between Method 1 and Method 2 was 2.7 (median = 3). As the values of *C*(*S*_*A*_, *S*_*B*_) can range between 0 and 100, this represents a mean difference of approximately 3 percentage points. The mean differences when comparing Method 1 with Method 3 and when comparing Method 1 with Method 4 were slightly higher (3.8 and 4.8, respectively). The standard deviations of the differences were also quite small, ranging from 1.9 (Method 1 versus Method 2) to 3.0 (Method 1 versus Method 4). This suggests that, while the choice of method for calculating the *C*(*S*_*A*_, *S*_*B*_) values makes some difference, it does not markedly change the measured degree of phosphorylation site conservation among the different species.

**Table 1:** Comparison between the values of *C*(*S*_*A*_, *S*_*B*_) generated using Method 1 and those generated using the other three methods (*X* = 2, 3, and 4).

	Degree				Directionality			
Method # (*X*)	Mean	Median	Range	Sthev	Mean	Median	Range	Sthev
2	2.7	3	[0, 19]	1.9	2.1	2	[−12, 19]	2.6
3	3.8	4	[0, 18]	2.6	0.8	1	[−11, 18]	4.5
4	4.8	5	[0, 26]	3.0	1.9	3	[−12, 26]	5.3

See the Materials and Methods for descriptions of each method. The “degree” section of the table represents the degree to which the values of *C*(*S*_*A*_, *S*_*B*_) obtained using Method 1 differ from those obtained using Methods 2, 3, and 4, and is calculated as |*C*_1_(*S*_*A*_, *S*_*B*_)−*C*_*X*_(*S*_*A*_, *S*_*B*_)|. The “directionality” section represents whether Method 1 tends to indicate more or less phosphorylation site conservation between pairs of species than the other methods (a positive mean and median indicates greater conservation, and vice versa), and is calculated as *C*_1_(*S*_*A*_, *S*_*B*_)−*C*_*X*_(*S*_*A*_, *S*_*B*_).

The four methods were also compared in order to measure the direction of differences between the *C*(*S*_*A*_, *S*_*B*_) values. This was done using a simple modification of the above procedure: to compare Method 1 with Method 2, *C*_1_(*S*_*A*_, *S*_*B*_)−*C*_2_(*S*_*A*_, *S*_*B*_) was calculated for each instead of |*C*_1_(*S*_*A*_, *S*_*B*_)−*C*_2_(*S*_*A*_, *S*_*B*_)|, and similarly for Method 1 versus Method 3 and Method 1 versus Method 4. The results of this analysis are also shown in Table 1.

Interestingly, the mean conservation among species was greater when using Method 1 (which used the full dataset of known human phosphorylation sites) than when using Method 2 (which used only known human phosphorylation sites that have been verified using low-throughput biological techniques). This is inconsistent with some prior findings, which have suggested that phosphorylation sites with unknown functions are less likely to be conserved [9, 10]. An initially plausible explanation for this observation is that there are different levels of intra-proteome redundancy of high-throughput sites as compared to low-throughput sites. Unfortunately, because of the method DAPPLE uses to predict phosphorylation sites, this cannot explain the discrepancy. Specifically, DAPPLE uses a BLAST search to determine the best match between a given human phosphorylation site (represented as a 15-mer peptide with the phosphorylated residue in the center) and the target proteome. For the purposes of this study, only the best match in a particular proteome is important—any other matches are ignored. This means that the result will be the same whether the target proteome contains just one match, or (say) 10 matches, making the level of intra-proteome redundancy irrelevant.

Determining phosphorylation site conservation by counting only non-conservative substitutions rather than all substitutions had a relatively small effect on the measured levels of phosphorylation site conservation (Method 1 versus Method 3; Table 1), although the standard deviation of the differences (4.5) was somewhat higher than for the comparison between Method 1 and Method 2. The mean difference when comparing Method 1 and Method 4, which differed both in the phosphorylation site dataset used and in the function for determining whether a phosphorylation site is present in a given species, was similar to the comparison between Method 1 and Method 2, although the standard deviation was higher, which was expected given that two variables were modified in the comparison between Method 1 and Method 4 versus only one in the comparison between Method 1 and Method 2.

S6–S8 Tables contain the values of *C*(*S*_*A*_, *S*_*B*_) obtained for all 432 species when using Method 2, Method 3, and Method 4, respectively; similarly, S2–S4 Figs contain the dendrogram for all 432 species when using Methods 2–4.

### Measuring protein kinase conservation

The degree of protein kinase conservation for the 20 species described earlier is shown in the lower triangle of Fig 1. Consistent with the hypothesis given at the end of the Introduction section, the degree of protein kinase conservation was generally greater than the degree of phosphorylation site conservation. For example, for *H. sapiens* and *M. musculus*, *C*(*S*_*A*_, *S*_*B*_) = 88 for phosphorylation sites and *C*(*S*_*A*_, *S*_*B*_) = 99 for protein kinases. The difference in conservation appeared to heighten at greater evolutionary distances; for instance, for *H. sapiens* and *I. scapularis*, *C*(*S*_*A*_, *S*_*B*_) = 20 for phosphorylation sites versus *C*(*S*_*A*_, *S*_*B*_) = 65 for protein kinases. However, there were a few exceptions to this trend, typically when one species in a pair was a plant. For the pair *A. thaliana* and *A. gambiae*, for example, *C*(*S*_*A*_, *S*_*B*_) = 27 for both phosphorylation sites and protein kinases.

### Measuring general protein conservation

**Fig 3:**
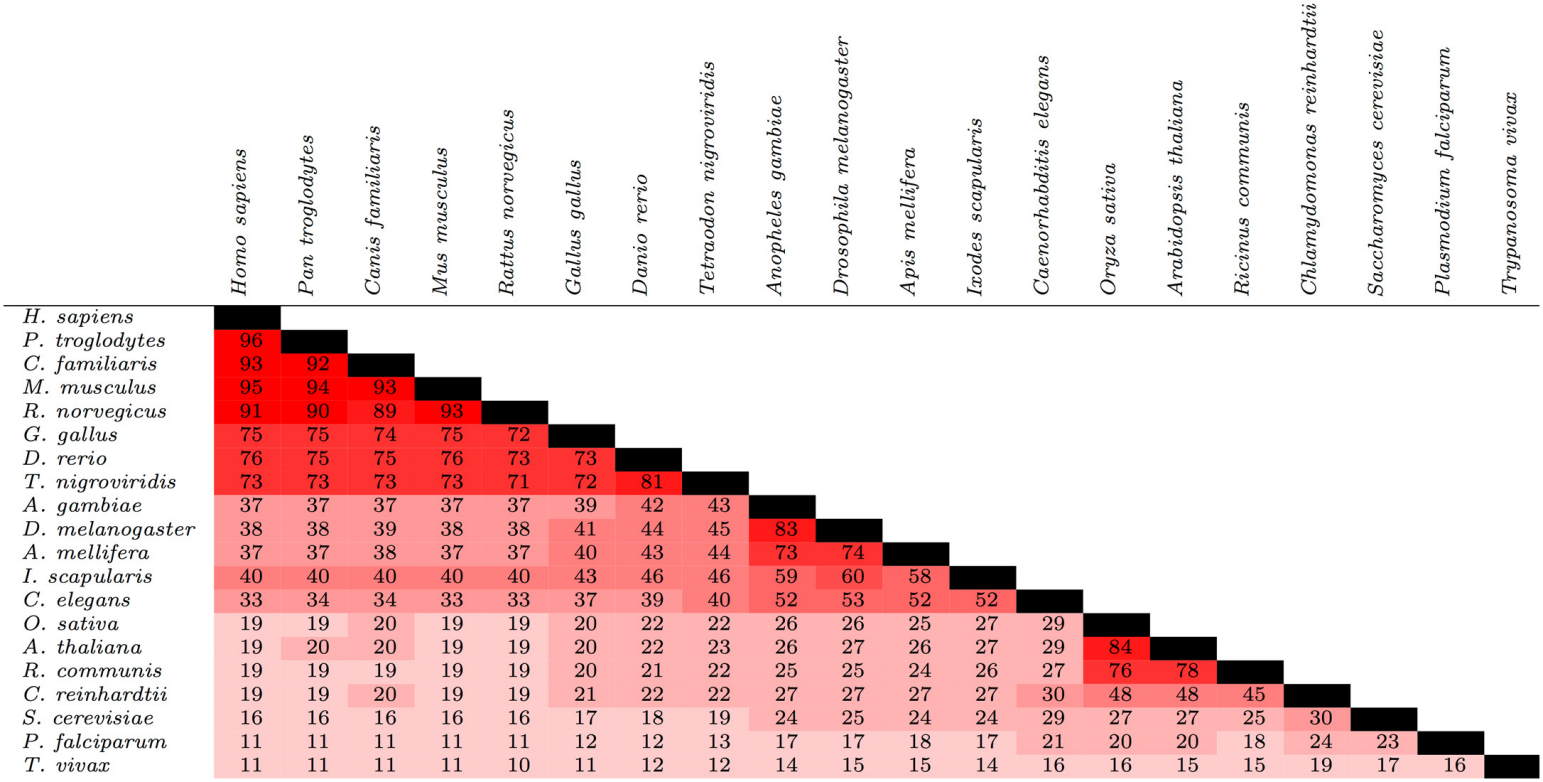
Conservation of proteins among 20 species from a diverse range of lineages. For a given pair of species, the values represent the percentage of proteins found in at least one of the two species that were found in both species. The cells are colored based on the value within; the closer the value is to 100, the brighter the shade of red.

The degree of conservation of proteins in general among the 20 species is shown in Fig 3. Overall, the degree of conservation of general proteins appeared to be higher than phosphorylation sites but lower than protein kinases. For instance, between *H. sapiens* and *M. musculus*, *C*(*S*_*A*_, *S*_*B*_) = 95 for general proteins, compared to 88 for phosphorylation sites and 99 for protein kinases. This trend appeared to extend to more distantly-related species; for instance, *C*(*S*_*A*_, *S*_*B*_) = 40 for general proteins between *H. sapiens* and *I. scapularis*, compared to *C*(*S*_*A*_, *S*_*B*_) = 20 and *C*(*S*_*A*_, *S*_*B*_) = 65 for phosphorylation sites and protein kinases, respectively. As before, pairs that included a plant were often exceptions to this pattern; for instance, *C*(*S*_*A*_, *S*_*B*_) = 26 for *A. thaliana* and *A. gambiae* versus 27 for both phosphorylation sites and protein kinases.

## Discussion

All eukaryotic species—even those that are closely related—exhibit substantial phenotypic differences. While some of these differences can be explained by the presence/absence of, or sequence variation within, specific genes and their corresponding proteins, regulatory mechanisms (e.g., post-translational modifications, DNA methylation, mRNA silencing and degradation, alternative splicing) also play a significant role. Phosphorylation is the most widespread post-translational modification in eukaryotes, and is integral to the control of almost every cellular signaling process. Thus, phosphorylation patterns dramatically affect the phenotype of a given organism. There are two major mechanisms by which phosphorylation-mediated regulatory pathways may be modified: the gain or loss of phosphorylation sites, and the gain or loss of protein kinases (although other mechanisms exist, such as sequence or structural changes to protein kinases that alter their specificity). We hypothesized that phosphorylation sites would be less conserved than protein kinases because less genomic modification is required for their gain or loss.

The data presented here support this hypothesis. For a given pair of species, the percentage of phosphorylation sites found in at least one of the two species that were found in both was generally less than the percentage of protein kinases found in at least one of the two species that were found in both (Fig 1, S1 Table and S6–S8 Tables). These data tentatively suggest that the gain or loss of phosphorylation sites may play a greater role in contributing to phenotypic differences among species than the gain or loss of protein kinases, and may help explain (for instance) why organisms with similar kinomes exhibit very different phenotypes.

While there exists a generally accepted taxonomy for eukaryotes, there is no definitive method for ascertaining phylogenetic relationships. Methods using genetic information, while widely used and accepted, can nonetheless exhibit significant biases [33]. Nonetheless, the tree we generated based on phosphorylation site conservation was very similar to the classifications given by the NCBI Taxonomy Browser [30], which amalgamates taxonomic information from multiple sources (Fig 2).

Although the results presented here provide considerable insight into the conservation of human phosphorylation sites in different species, certain limitations should be noted. First, the existence or non-existence of a given non-human site was predicted using sequence homology to known human sites. While sequence homology is well-established as a powerful predictor of structure and function, the fact that the sequence surrounding a known phosphorylation site in one organism is conserved in a second organism does not guarantee that the site is actually phosphorylated in the second organism. Phosphorylation site prediction is a difficult problem, and the accuracy of even the best predictors is limited [18–20]. However, it should also be noted that previous assessments of classifier accuracy were based on the goal of predicting completely novel phosphorylation sites (that is, sites that are not homologous to some other site) within the same organism. This is more difficult than the problem addressed here, which is using phosphorylation sites that are already known to exist in one organism (human in this study) to identify homologous sites in other organisms. Second, it should be emphasized that all of the known phosphorylation sites used in this study were from human; thus, this study characterizes only the evolution of known *human* phosphorylation sites, and does not capture the conservation of phosphorylation sites that are not found in human. It has been found that some species contain entire classes of protein kinases that are absent from other species; for example, around a third of the protein kinases in the proteome of *P. falciparum* belong to a class of protein kinases called FIKK (so-called because a conserved motif in these kinases contains the amino acid segment phenylalanine-isoleucine-lysine-lysine), which appear to be largely absent from other eukaryotic kinomes [34]. The conservation of sites whose phosphorylation is catalyzed by such kinases would not be reflected in the current analysis. Third, our definition of site conservation does not make any assumptions about the function of a site. Sequence conservation is a necessary, but not sufficient, condition for phosphorylation to occur. Thus, it is likely that some of the sites for which the sequence is conserved are not functional. Interestingly, however, Freschi et al. [7] estimated that of the phosphorylation sites that are known to exist in either human or mouse (but not both) and that are conserved at the sequence level, 95% are likely to be functionally phosphorylated in the other species. While it is possible that this percentage may differ depending on the relatedness of the species being compared, it nonetheless gives considerable confidence in our sequence similarity-based method for estimating phosphorylation site conservation.

In summary, we would like to emphasize that, given the predictive nature of this study, additional research—preferably using experimentally-determined phosphorylation sites—is required to more clearly characterize phosphorylation site conservation among species, and to define the relative importance of phosphorylation site evolution and protein kinase evolution in influencing phenotype. As more and more phosphorylation sites are discovered in different organisms using mass spectrometry and other biological techniques, our ability to accurately characterize phosphorylation site conservation will continue to improve.

## Supporting Information

S1 Table Values of *C*(*S*_*A*_, *S*_*B*_) for all 432 species when using Method 1. (XLSX)

S2 Table Values of *C*(*S*_*A*_, *S*_*B*_) for all 432 species for Ser sites when using Method 1. (XLSX)

S3 Table Values of *C*(*S*_*A*_, *S*_*B*_) for all 432 species for Thr sites when using Method 1. (XLSX)

S4 Table Values of *C*(*S*_*A*_, *S*_*B*_) for all 432 species for Tyr sites when using Method 1. (XLSX)

S5 Table Average value of *C*(*S*_*A*_, *S*_*B*_) for each species (averaged over all of the other species) for each phosphorylated residue when using Method 1. (XLSX)

S6 Table Values of *C*(*S*_*A*_, *S*_*B*_) for all 432 species when using Method 2. (XLSX)

S7 Table Values of *C*(*S*_*A*_, *S*_*B*_) for all 432 species when using Method 3. (XLSX)

S8 Table Values of *C*(*S*_*A*_, *S*_*B*_) for all 432 species when using Method 4. (XLSX)

S1 Fig Dendrogram created using hierarchical clustering for all 432 species when using Method 1. (PDF)

S2 Fig Dendrogram created using hierarchical clustering for all 432 species when using Method 2. (PDF)

S3 Fig Dendrogram created using hierarchical clustering for all 432 species when using Method 3. (PDF)

S4 Fig Dendrogram created using hierarchical clustering for all 432 species when using Method 4. (PDF)
