# Supplementary figures and images for: Computational Analysis of the Predicted Evolutionary Conservation of Human Phosphorylation Sites
Source: PLoS One. 2016 Apr 5;11(4):e0152809. doi: 10.1371/journal.pone.0152809 (PMC4821552; doi:10.1371/journal.pone.0152809)

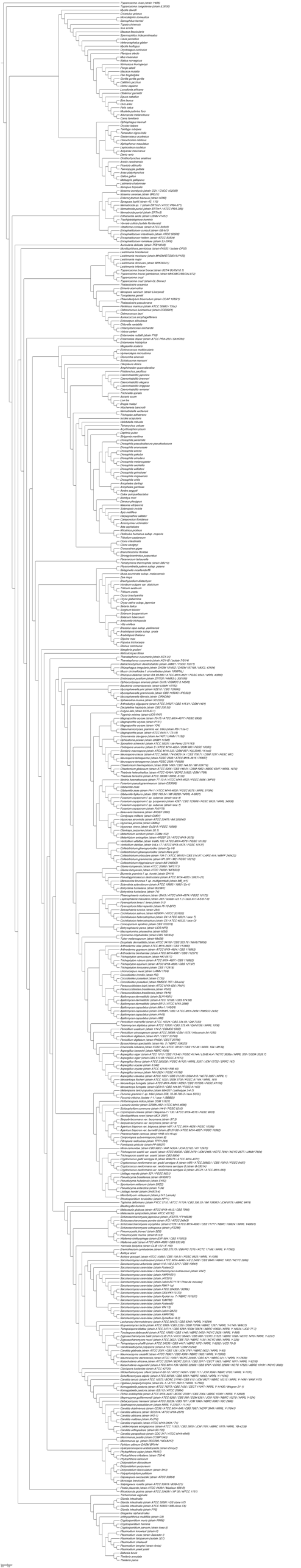

Supplement: S1 Fig — (PDF) [file pone.0152809.s009.pdf]

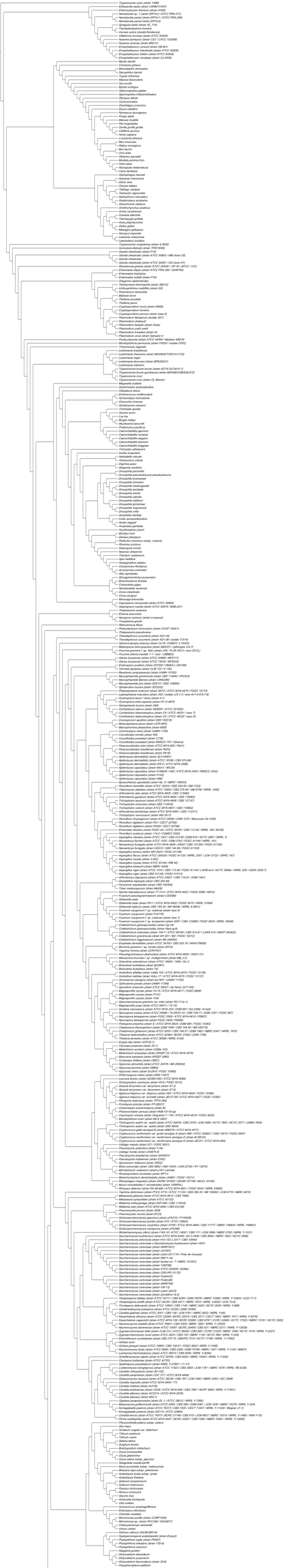

Supplement: S2 Fig — (PDF) [file pone.0152809.s010.pdf]

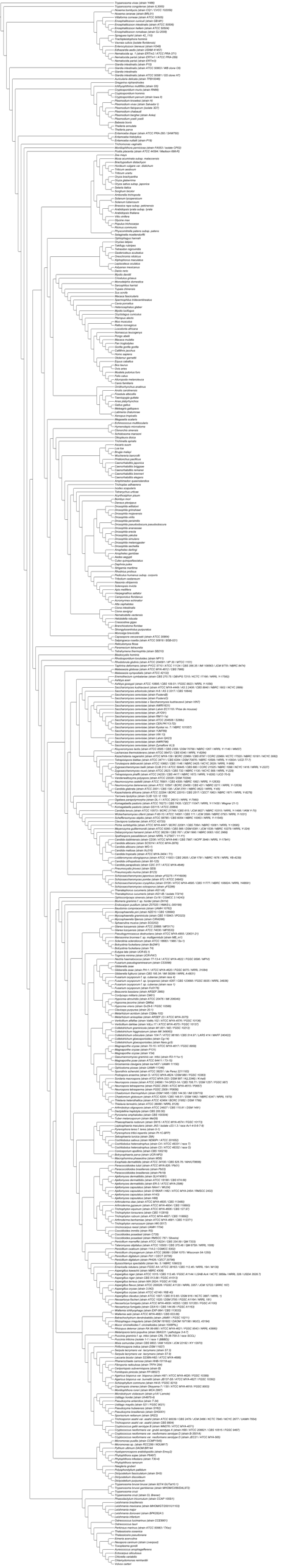

Supplement: S3 Fig — (PDF) [file pone.0152809.s011.pdf]

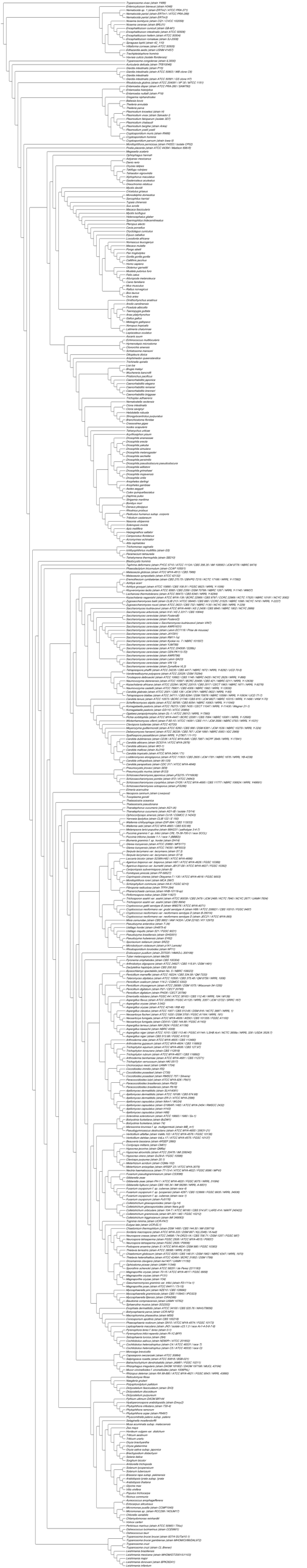

Supplement: S4 Fig — (PDF) [file pone.0152809.s012.pdf]
